# Role of Empathy and Lifelong Learning Abilities in Physicians and Nurses Who Work in Direct Contact with Patients in Adverse Working Conditions

**DOI:** 10.3390/ijerph19053012

**Published:** 2022-03-04

**Authors:** Roberto C. Delgado Bolton, Montserrat San-Martín, Luis Vivanco

**Affiliations:** 1 Department of Diagnostic Imaging (Radiology) and Nuclear Medicine, University Hospital San Pedro and Centre for Biomedical Research of La Rioja (CIBIR), 26006 Logroño, Spain; rcdelgado@riojasalud.es; 2 Platform of Bioethics and Medical Education, Centre for Biomedical Research of La Rioja, 26006 Logroño, Spain; 3 National Centre of Documentation on Bioethics, Rioja Health Foundation, 26006 Logroño, Spain; 4 Department of Statistics and Operational Research, University of Granada, 52003 Melilla, Spain; momartin@ugr.es; 5 Faculty of Health Sciences, European Atlantic University, 39011 Santander, Spain

**Keywords:** somatization, exhaustion, work alienation, empathy, lifelong learning, nursing, medicine

## Abstract

Empathy and lifelong learning are two professional competencies that depend on the four principles of professionalism: humanism, altruism, excellence, and accountability. In occupational health, there is evidence that empathy prevents work distress. However, in the case of lifelong learning, the evidence is still scarce. In addition, recent studies suggest that the development of lifelong learning varies in physicians and nurses and that it is sensitive to the influence of cultural stereotypes associated with professional roles. This study was performed with the purpose of determining the specific role that empathy and lifelong learning play in the reduction in occupational stress. This study included a sample composed by 40 physicians and 40 nurses with high dedication to clinical work in ambulatory consultations from a public healthcare institution in Paraguay. Somatization, exhaustion, and work alienation, described as indicators of occupational stress, were used as dependent variables, whereas empathy, lifelong learning, gender, discipline, professional experience, civil status, and family burden were used as potential predictors. Three multiple regression models explained 32% of the variability of somatization based on a linear relationship with empathy, lifelong learning, and civil status; 73% of the variability of exhaustion based on a linear relationship with empathy, somatization, work alienation, and discipline; and 62% of the variability of work alienation based on a linear relationship with lifelong learning, exhaustion, and discipline. These findings indicate that empathy and lifelong learning play important roles in the prevention of work distress in physicians and nurses. However, this role varies by discipline.

## 1. Introduction

### 1.1. Adverse Working Condition in Clinical Settings

The concept of “adverse working conditions” has been associated with harsh working environments, where resources are either scarce or inadequately distributed, keeping the daily work an odyssey [1]. In healthcare settings, these environments usually combine three aspects [2]: (a) lack of resources (insufficient, poorly managed, or misused); (b) lack of recognition (or support) from institutions, supervisors, or co-workers; and (c) prevalence of an organizational culture that places institutional benefits above patients. Paraguay, similar to other low-to-middle income countries (LMICs), has high indexes of social inequity and corruption, a phenomenon reported as a prevalent social problem affecting the majority of Latin American healthcare systems [3]. Although important efforts have been made in Paraguay’s public health system to try to improve this situation, the country still has one of the poorest healthcare indicators in all of Latin America [4]. Not only patients but also physicians and nurses working in these public healthcare institutions suffer the negative consequences derived from the adverse working conditions mentioned above. Before the COVID-19 pandemic started in 2020, the General Division of Statistics, Surveys, and Census of Paraguay calculated that 20% of Paraguayan families had unsatisfied health needs [5]. In addition, another study reported that 73% of the patients with few economic resources have to pay for their own treatments [6]. These out-of-pocket expenses were mainly associated with medications, diagnosis and laboratory expenses, medical consultations, and hospitalizations. In fact, according to the PAHO’s report [7], half of the total health expenditure in Paraguay comes from out-of-pocket expenditures. In terms of human resources in health, Paraguay has one of the lowest rates of physicians and nurses by inhabitants in all of Latin America: two physicians and four nurses for every 10,000 inhabitants, while the means in all of Latin America are 22 and 46, respectively [7]. These working conditions can explain the high prevalence of burnout in Paraguayan physicians, especially in specialties with high social demand [8], or the high prevalence of job dissatisfaction among nurses and other healthcare professionals [9]. In nurses, this issue is even more sensitive, taking into consideration that, due to their work duties, they are more exposed to public complaints in their daily work.

### 1.2. Empathy and Lifelong Learning as Components of Professionalism in Medicine and Nursing

Louise Arnold and David Stern defined professionalism in healthcare disciplines, such as medicine as nursing, as a foundation of clinical competence, communication skills, and ethical and legal understanding, upon which the aspiration to and wise application of its four basic principles is built: excellence, humanism, accountability, and altruism [10]. According to Arnold [11], empathy and lifelong learning are two abilities that should be considered specific components of this professionalism. 

In the case of empathy, it is described as a central attribute of the *humanism* and *altruism* in both disciplines [11,12]. Solid research evidence demonstrates its importance in the improvement of clinical outcomes and patients’ satisfaction [13]. In addition, recent studies indicate that more empathetic physicians [12,14,15], and nurses [12,15,16] suffer less burnout than those who are not. Based on this, some authors suggest that leaders and educators in medical and nursing schools and in healthcare institutions should consider enhancing and sustaining empathy in healthcare professional education and practice, respectively [17]. Research evidence supporting this shows that empathy is an attribute that is amenable to change with a targeted training and educational intervention due to a more cognitive than emotional nature. This attribute can be summarized by three main aspects [13]: (i) the capacity to understand patients’ needs and concerns related to their disease and treatment; (ii) the capacity to establish an adequate communication with the patients, even when a language barrier is visible; and (iii) the desire to help. In fact, based on this conceptual framework, several authors agree that an emotional response does not play the main role in an empathic response in clinical encounters [18,19,20,21]. This distinction acquires especial relevance, taking into account that in healthcare institutions, physicians, and nurses suffer daily exposure to situations with a high emotional load, which can be derived from greater emotional exhaustion, stress, and burnout if their emotions become out of self-control [16,22]. Additionally, in working conditions with scarce resources, excessive patient workload, or patients unable to pay for their treatments, as usually occurs in LMICs, it is expected that being emotionally involved can be risky rather than beneficial [23]. Unfortunately, an enhancement in empathy either in medical and nursing education or in healthcare institutions is a pending task in the majority of countries, especially in those where resources are scarce [24,25,26].

In the case of lifelong learning, Arnolds identified it as an expression of the other two basic principles of professionalism: *excellence* and *accountability* in health [11] (p. 503). Lifelong learning in clinical settings has been described as a set of self-initiated activities (a behavioural aspect) and information-seeking skills (capabilities) that are activated in individuals with a sustained motivation (predisposition) to learn and the ability to recognize their own learning needs (a cognitive aspect) [27]. This ability has been widely supported by medical and nursing educators. It is expected that lifelong learning can play a similar role as empathy in the occupational health and well-being of both groups of healthcare professionals. A recent study performed in Latin American hospitals has reported an inverse correlation between lifelong learning and measures of somatization and work alienation in physicians from Colombian, Mexican, Ecuadorian, and Argentinean institutions [23], similar to the ones reported between empathy and self-perception measures of somatization, exhaustion, and work alienation in Spanish physicians and nurses [15]. However, evidence reported in LMICs is not always consistent with differences by discipline [23,28] and by the working environment [2,29]. Imbalanced access in lifelong learning activities, a lack of social support at the workplace, and a mismatch between efforts and rewards are usually described as causes of the limited impact that lifelong learning abilities have in healthcare professionals in Latin American [2,23,30] and in African countries [28,29]. An imbalanced access to lifelong learning activities has been reported in working environments where medicine is set above nursing or in societies where nursing has low job control in comparison with medicine [15,23,28]. Attitudes toward lifelong learning are enhanced in workplaces where workers have high job control and receive social support from their colleagues, superiors, or institutions [29]. There is evidence supporting the positive role that social support plays, for example, in the improvement of nurses’ attitudes toward lifelong learning abilities, in work engagement, and in the prevention of work distress [28,29,31,32]. The Job–Demand–Control Social Support model (JDCS model), initially proposed by Karasek and Theorell [1], offers a theoretical framework that explains this effect. According to this model, high job demands, low job control, and low social support additively predict workers’ high stress reactions, which finally affect their working performance and motivation to learn. Finally, the Effort–Reward Imbalance model (ERI model) can explain the effect that the mismatch between efforts and rewards has in the working life of physicians and nurses. According to the ERI model [33], professionalism is supported on a contract of social reciprocity, where colleagues; supervisors; institutions; patients; and the society, as a whole, can provide rewards not only in economic terms but also in personal acknowledgement or professional recognition. A lack of those rewards can be detrimental to the development of professionalism, especially among professionals who strive to do good work under adverse working conditions [2]. 

### 1.3. Study Purpose

Recently, an observational study performed with Bolivian physicians demonstrated that abilities toward inter-professional collaboration explained variations in the scores of exhaustion in physicians working in a main public hospital of La Paz [2]. This ability, jointly with empathy and lifelong learning, has been described as a specific component of professionalism. On this basis, this study was designed with the main purpose of determining the role that empathy and lifelong learning abilities, the other two specific components of professionalism, play in the prevention and management of work-related stress in Paraguayan physicians and nurses who are in direct contact with patients, such as ambulatory consultations. The following hypothesis was tested and confirmed: empathy and lifelong learning play roles that influence the prevention of occupational stress even under adverse working circumstances. However, in clinical settings in which physicians assume the main responsibility of patients’ treatment and carry the main emotional burden of clinical decisions, this effect could be greater among them in comparison with among nurses. Four research objectives, aligned with the aforementioned hypothesis and the purpose of this Special Issue, were set:

(i) To measure empathy and lifelong learning as potential predictors of greater occupational well-being and as symptoms associated with work distress in a sample of Paraguayan physicians and nurses working in direct contact with patients in ambulatory consultations;
(ii) To compare the abovementioned measures by discipline in order to confirm possible differences;
(iii) To confirm whether work distress measures correlate with empathy and lifelong learning in the entire sample and in physicians’ and nurses’ sub-groups; and
(iv) To confirm whether empathy and lifelong learning play roles that influence lower self-perception of symptoms related to work distress.

## 2. Methods

### 2.1. Participants

A sample of 80 healthcare professionals, 40 physicians and 40 nurses, in ambulatory consultations from the departments of family medicine, general surgery, paediatrics, gynaecology and obstetrics, and nephrology from the Regional Hospital of Concepcion in Concepcion City, Paraguay, participated in this study. These departments presented the highest demand of patients of the abovementioned institution. All participants were healthcare professionals directly contracted by the referred institution with full dedication to patients’ care. Healthcare professionals in training were not included in this study. The estimation of the sample size required for this study was calculated with G*Power software, version 3.1.9.6. This calculation was carried out by taking into consideration the creation of three separate regression models for somatization, exhaustion, and work alienation. Each regression model was based on a linear multiple regression analysis with an effect size between medium and high (Cohen-*f*^2^ = 0.30), with an alpha equivalent to 0.05, with a power of 0.95, and with at least three tested predictors from 10 variables analysed. It was also assumed that 20% missing questionnaires were missing (questionnaires that were partially answered). According to this analysis, the minimum sample size should be 78 participants.

The study design, approved by an independent ethical committee (Ref. CEICLAR-PI-199), was carried out in accordance with the recommendations and authorization of the participating institution’s administration (1ra Región Sanitaria de Salud, Ministerio de Salud Pública y Bienestar Social de Paraguay). All healthcare professionals who agreed to participate in this study provided written informed consent. Each participant received a paper-form questionnaire provided together with an information letter in an enclosed envelope that was returned to a local researcher once they responded, following the abovementioned protocol, previously approved. Participation by healthcare professionals was anonymous and voluntary. 

### 2.2. Main Measures

Self-perception of somatization, exhaustion, and work alienation were used as the main measures. The Scale of Collateral Effects (SCE) of the Questionnaire of General Labour Well-being was administered as the measuring instrument [34]. The SCE is composed by three mini-scales: the scale of somatization (SS), with five items; the scale of exhaustion (SE), with four items; and the scale of work alienation (SA), with four items. Each item of the abovementioned mini-scales starts with the following statement: “*Currently, because of my work, I feel:*” followed by one specific symptom. The SS measures the following symptoms: digestive disorder, back pain, insomnia, headache, and muscle tension. Symptoms in the SE included work overload, emotional exhaustion, physical exhaustion, and mental saturation. Finally, the SA assesses bad mood, low personal fulfilment, depersonalized treatment, and frustration. The perception of each symptom is answered following a seven-point Likert-type scale reflecting a daily frequency in the last week from 1 (never) to 7 (always). The original version of the SCE was developed in Spanish [34]. This instrument was originally tested in professionals from different disciplines in Spain and in Latin American countries showing good psychometric properties with alpha coefficients of 0.91, 0.87, 0.87, and 0.82, for the global score (SCE) and the scores of somatization (SS), exhaustion (SE), and work alienation (SA), respectively [34]. In the context of medicine and nursing, this instrument has been previously proven in Spanish [15] and Latin American [2,23] healthcare institutions with excellent results.

In addition, the Jefferson Scale of empathy (JSE) [25] and the Jefferson Scale of physician’s lifelong learning (JeffSPLL) [35], instruments designed for measuring two specific components of medical professionalism in healthcare professionals, were used. The JSE was developed based on the definition of empathy as a cognitive attribute that involves an understanding of the inner experiences and perspectives of the patient, combined with a capability to communicate this understanding to the patient. The 20 items in the JSE are grouped among three factors: “perspective taking”, “compassionate care”, and “walking in the patient’s shoes” [36]. Each item of the JSE is answered on a seven-point Likert scale from 1 (strongly disagree) to 7 (strongly agree). A sample item of the JSE is “I try to understand what is going on in my patients’ minds by paying attention to their nonverbal cues and body language”, which corresponds with the “perspective taking” domain. The JeffSPLL measures the skills shown by the healthcare professional in searching for information, in self-motivating, and in taking advantage of learning opportunities. The JeffSPLL was developed based on a definition of lifelong learning as a concept involving a set of self-initiated activities (behavioural aspects) and information-seeking skills (capabilities) that are activated in individuals with a sustained motivation (predisposition) to learn and the ability to recognize their own learning needs (cognitive aspect) [36]. The 14 items of the JeffSPLL are distributed among three factors: “beliefs and motivations in learning”, “attention to learning opportunities”, and “skills in the search for information”. Each item of the JeffSPLL is answered on a four-point Likert scale from 1 (strongly disagree) to 4 (strongly agree). A sample item of the JeffSPLL is “I believe that I would fall behind if I stopped learning about new developments in my profession”, which corresponds with the domain of “beliefs and motivations in learning”. In the aforementioned scales, higher scores are associated with greater development of the elements measured. The Spanish versions of the JSE and the JeffSPLL have been validated and tested with Spanish and Latin American healthcare professionals showing consistent psychometric properties [23,25].

Finally, in a socio-demographic form, other seven variables were collected: age (years), gender (male or female), civil status (single, married, or other), family burden (number of family members economically dependent of the respondent), discipline (medicine or nursing), professional experience (years), and salary range (a monthly salary equal or higher than USD 1000, or a monthly salary lower than USD 1000). 

### 2.3. Statistical Analysis

Following international recommendations [37], only psychometric measures with alpha coefficients equal or higher than 0.70 were included in the analyses. Scales of somatization, exhaustion, and work alienation were used as dependent variables. After normality was assessed, using Pearson’s chi-squared and Lilliefors–Kolmogorov–Smirnov tests, comparative analyses using non-parametric Mann–Whitney U tests were performed to determine differences in the abovementioned scales by discipline. Effect size (*r*) was calculated following the formula described by Fritz, Morris, and Richler [38] and by Tomczak and Tomczak [39] for non-parametric tests. Following the recommendation of Hojat and Xu [40], an *r*-value equal to 0.50 was considered a large effect size with crucial practical importance; an *r*-value equal to 0.30 was considered a medium effect size, with moderate practical importance; and an *r*-value equal to 0.10 was considered a small effect size, with negligible practical importance.

Regarding age, family burden, and global scores on empathy and lifelong learning, Spearman’s correlation analyses were performed in order to determine statistical associations between them and scores on somatization, exhaustion, and work alienation. Separate multiple linear regression analyses were performed using scores on somatization, exhaustion, and work alienation as dependent variables, while all others were used as potential predictors. A regression model was accepted only if conditions of statistical inference (normality, zero mean, constant variance, and uncorrelatedness of the residuals, in addition to linearity and absence of multi-collinearity) were met. In order to quantify the degree of practical significance of a model obtained, the effect size (Cohen’s-*f*^2^) was calculated: a value equal to or greater than 0.02, and smaller than 0.15 was interpreted as a small effect; a value equal to or greater than 0.15, and smaller than 0.35 was interpreted as a medium effect; and a value equal to or greater than 0.35 was interpreted as a large effect, following the interpretation criteria proposed by Cohen [41]. 

All analyses were performed using R statistical software, version 4.1.1., for Windows. The statistical analyses of the data also included the “multilevel” [42], “rstatix” [43], “lsr” [44], and “nortest” [45] packages.

## 3. Results

The average age of the sample was 36 (*SD =* 7) years old, with a range between 24 to 51 years old. By gender, 31 (39%) were male and 49 (61%) were female. By civil status, 41 (51%) were single while 39 (49%) were married. By family burden, 15 (19%) participants indicated not experiencing family burden, while the other 65 (81%) indicated having from one to nine people economically dependent on them. By salary range, 38 (47.5%) had monthly salaries equal to or higher than USD 1000, while the other 42 (52.5%) had monthly salaries lower than USD 1000. By professional experience, 44 (55%) had five or less years of professional experience, while 36 (45%) had more than five years of professional experience working with patients. 

With regard to the first research objective related to the measurement of work distress (SCE), empathy (JSE), and lifelong learning (JeffSPLL), all scales showed adequate psychometric properties with values similar to the ones previously reported in other studies with healthcare professionals from other Latin American countries [2,16,23,25,30]. The score distribution, descriptive statistics, and reliability for the instruments used in this study are described in Table 1. 

**Figure 1:**
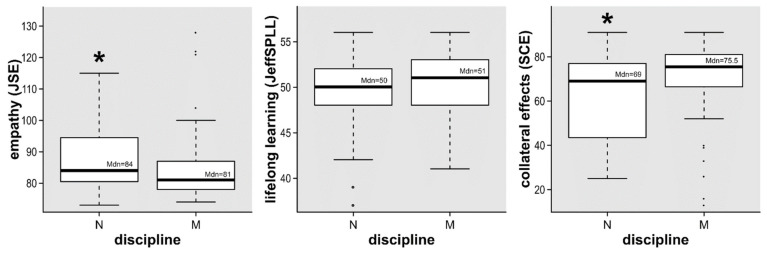
Global scores of empathy, lifelong learning, and collateral effects measures in a sample of healthcare professionals. JSE, Jefferson Scale of Empathy; JeffSPLL, Jefferson Scale of physician lifelong learning; SCE, Scale of collateral Effects; M, medicine; N, nursing; * *p* < 0.05.

With regard to the second research objective related to professionalism and work distress perception by discipline, Mann–Whitney U tests confirmed a higher development on empathy in nurses (*p *= 0.04), with a small to medium size effect (*r *= 0.23), and a higher perception on collateral effects in physicians (*p *= 0.03), with a small to medium size effect (*r *= 0.24). Differences by discipline in lifelong learning abilities were not confirmed (*p *= 0.35). A summary of these findings is shown in Figure 1.

Concerning the third research objective related to correlation between collateral effects and professionalism, analyses confirmed an inverse correlation between empathy and the global score of collateral effects in the entire sample (ρ = −0.32; *p* = 0.004) and in the group of physicians (ρ = −0.34; *p* = 0.03). However, this correlation was not confirmed in the group of nurses (ρ = −0.25; *p* = 0.12). On the contrary, analyses confirmed a positive correlation between lifelong learning and the global score of collateral effects in the entire sample (ρ = +0.27; *p* = 0.02) and in the sample of physicians (ρ = +0.52; *p* < 0.001). In the group of nurses, this correlation was not confirmed (ρ = −0.05; *p* = 0.76). In addition, no correlation was observed between the global score of collateral effects and the variables age and family burden.

Based on these preliminary findings, three regression models using multiple linear regression analyses were created (fourth research objective), as shown in Table 2. In the first case, a model explaining the 32% of variance in somatization (R^2^-adjusted = 0.29; *F*_(3,76)_ = 11.7; *p* < 0.001), with a large effect size (Cohen-*f*^2^ = 0.46), was obtained. According to this model, being less empathetic (*p* < 0.001), having higher lifelong learning abilities (*p* < 0.001), and being single (*p *= 0.038) appeared to be predictors of greater somatization in healthcare professionals. In the second case, the model obtained explained the 73% variance in exhaustion (R^2^-adjusted = 0.72; *F*_(4,75)_ = 50.67; *p* < 0.001), with a large effect size (Cohen-*f*^2^ = 2.70). According to this model, being less empathetic (*p *= 0.047), scoring high in symptoms associated with somatization (*p* < 0.001) and work alienation (*p* < 0.001), and being a physician (*p *= 0.014) appeared to be predictors of greater exhaustion. Finally, a model explaining the 62% of variance in work alienation (R^2^-adjusted = 0.61; *F*_(3,76)_ = 41.44; *p* < 0.001), with a large effect size (Cohen-*f*^2^ = 1.64), was obtained. According to this model, having lower development in lifelong learning abilities (*p *= 0.01), scoring high in exhaustion (*p* < 0.001), and being a nurse (*p *= 0.001) appeared to be predictors of greater work alienation. The three above-mentioned models fulfilled all of the necessary conditions for statistical inference, confirming the hypothesis of this study. In addition, two separate linear regression analyses were performed using empathy and lifelong learning as dependent variables while all the others variables were used as potential predictors. These analyses were performed in order to explore whether empathy and lifelong were sensitive to the influence of the other variables collected. However, none of the models obtained fulfilled all conditions for statistical inference. 

## 4. Discussion

In the present study, our global scores on the SCE were higher than those previously reported in physicians and nurses from Spanish [15] and Latin American [23] public healthcare institutions, suggesting a greater self-perception based on measures of somatization, exhaustion, and work alienation in the study group. These findings are in accordance with the high prevalence of burnout previously reported in Paraguayan healthcare professionals [8]. On the contrary, the study sample showed very low scores on empathy, which were lower than those reported in studies with healthcare professionals from the United States [46], Italy [47], Korea [48], or Spain [25]. Furthermore, they were also lower than those reported in other Latin American countries [23,25], including one carried out in a Bolivian public hospital declared in an institutional emergency [2]. Additionally, these scores were inverse correlated with global scores on collateral effects, which is in accordance with the inverse association between empathy and work distress previously reported in the literature [14,15,23]. In the case of lifelong learning, the entire sample showed higher scores than those previously reported in Latin America [2,23], Spain [49], United States [35], Uganda [28], and Iran [50]. However, opposite to those for empathy, these scores were positively correlated with the global perception of collateral effects. These findings were consistent with the limited impact that lifelong learning has shown in other Latin American institutions [2,23].

### 4.1. Empathy and Lifelong Learning and Their Role in the Prevention of Occupational Stress

The primary aim of this study was to characterize the specific role that empathy and lifelong learning abilities play in relation to occupational well-begin in healthcare professionals, measured from the scope of somatization, exhaustion, and work alienation at the workplace. In the first case, a regression model based on three predictors explained 32% of the variance of somatization. According to this model, empathy and being married appeared to be two negative predictors of somatization, while lifelong learning appeared to be a positive one. These findings are in consonance with previous studies where empathy has shown a protective role in the prevention of work distress and burnout in healthcare professionals [14,15,23] and in medical students [51]. In addition, married workers who participated in this study reported less somatization than their single colleagues. This finding is consistent with another study where Spanish healthcare professionals suffering family and romantic loneliness reported greater symptoms of somatization [15]. Furthermore, in the abovementioned study, family burden was not associated with greater symptoms of somatization among those workers. Therefore, this finding brings new evidence supporting the important role that family life plays in the prevention of work distress. This evidence suggest that having a family life offers, as a whole, an important source of social support, healthy distraction, and personal fulfilment that help workers deal with the daily difficulties at their workplace. Regarding lifelong learning, the findings observed in this study help to clarify the exactly role that this ability plays in relation to somatization. In previous studies with Latin American healthcare professionals, lifelong learning played a protective role only in the case of physicians and in working environments with better conditions than the one described in this study [23]. By contrast, in nurses and in physicians who work in adverse working conditions, a correlation between lifelong learning and somatization was not confirmed in previous studies [2,23]. The findings reported in this study indicate that, in harsh working environments, greater development in learning abilities adds extra physical and emotional effort to the daily work overload, which is probably not rewarded. This is consistent with the phenomenon conceptualized by the ERI-model [33].

In the second case, four predictors explained 73% of the variance on exhaustion. Somatization and work alienation play additive influential roles on the perception of symptoms related to exhaustion. This effect is consistent with the theoretical conceptualization of collateral effects at the workplace, initially described by Blanch et al. [34]. Furthermore, being a nurse and being more empathic with the patients reduce the risk of suffering exhaustion at the workplace. This protective role, in the case of empathy, has been previously described in other studies with Latin American and Spanish healthcare professionals [15,23]. According to the model obtained, nurses suffer less exhaustion than their medical colleagues do. In Paraguay, as in other countries where medicine is set above nursing [23,26,52], physicians probably assume the main responsibility of patients’ treatment. Thus, it is expected that nurses are less exposed to the emotional burden that accompanies clinical decisions in comparison with physicians [16,23]. 

Finally, a regression model explained the 63% of variance in work alienation. In this model, similar to the one previously described, exhaustion plays an additive role following the conceptualization initially described by Blanch et al. [34]. However, according to this model, nurses were more exposed to suffering work alienation in comparison with physicians, while lifelong learning appeared to be a protective factor against work alienation. Different from the other two models, in this model, empathy did not appear to be protective factor. The reason for this can be the very low scores on this ability in the entire study sample. It can also explain why this lack of empathy especially affects nurses until the point that being a nurse becomes riskier for suffering work alienation in the study group. By contrast, being focused on lifelong learning keeps healthcare professionals connected and motivated in their work duties. These findings are consistent with those in which empathy has been demonstrated to be especially important in the prevention of work alienation in nurses [15]. 

Although the current emphasis on professionalism in disciplines such as medicine or nursing places high value on the enhancement of excellence, humanism, accountability, and altruism, represented by empathy and lifelong learning abilities, most students and healthcare professionals do not routinely acquire the skills needed to demonstrate them. In the frame of the Special Issue entitled “work organization, occupational stress, and mental health and wellbeing: advances in the evidence and approaches to intervention”, the findings reported in the present study highlight some issues that require special attention. First, an adverse working condition has demonstrated, with time, a negative effect on the mental health and wellbeing of physicians and nurses. Second, this effect is also detrimental in the maintenance of a professional performance, reflected in low scores in empathy and lifelong learning measures of professionals daily exposed to such environments. Third, this effect is not equal in physicians and in nurses due to the different professional roles that both groups play in patients’ healthcare. Finally and probably the most important, a greater development of empathy and lifelong learning abilities reduces the negative effect derived from working in such circumstances. In countries, such as Paraguay, where resources are scarce and organizational improvements imply efforts in different levels that are, in most of the cases, out of reach for health managers, supervisors, or workers, considering the enhancement of empathy and lifelong learning in healthcare professionals could be a goal that is capable of being reached. Evidence demonstrates, for example, that targeted training programs are effective in the improvement of empathy and lifelong learning abilities in students as well as in physicians and nurses in training and in practice [2,53,54,55,56]. To reduce the current trends in medical and nursing education and in practice, it is urgent to develop and implement tools oriented toward the acquisition, maintenance, and improvement of both abilities in the different stages of the professional lifespan of physicians and nurses, including undergraduate, graduate, and continuing education in schools and in hospitals. 

### 4.2. Limitations and Future Research Directions

The study was performed in a sample of healthcare professionals, physicians, and nurses with high dedication to patients’ consultations in a single hospital institution with high social demand. Therefore, the results should be interpreted with some caution. The findings reported in this study cannot be extrapolated to other medical services where contact with patients is limited. This is the case of technology-oriented specialities, where healthcare professionals perform highly skilled and specialized therapeutic techniques or procedures and where direct contact with the patients is null or limited (e.g., laboratory specialties, medical imaging specialties, ophthalmology, traumatology, and specific surgical services). 

Further studies with healthcare professionals working in those services are necessary in order to determine the role that empathy and lifelong learning abilities play in those settings. In addition, this study brings new evidence suggesting that personal life stage (measured by civil status and family burden) also influence how healthcare professionals cope with stressful situations in working environments with high social demand. Based on this evidence, future research work may focus on a deeper analysis of the role that the family environment plays in the reduction in occupational stress of these healthcare professionals. Finally, similar studies in other healthcare institutions of Paraguay and in healthcare institutions from other countries with similar sociocultural and economic characteristics could bring new evidence clarifying if the effect reported in this study is not limited to a specific institution.

## 5. Conclusions

All of these findings stress the importance that empathy and lifelong learning, two specific components of professionalism, have in healthcare professionals’ health and welfare. The findings reported in this study also acquire special practical importance, taking into account that empathy and lifelong learning have in common being predominant cognitive abilities (more than emotional), which can be trained and reinforced with adequate formative and supportive working strategies. This study also highlighted the role that cultural behaviours play in the interaction between professionalism and occupational well-being in healthcare professionals. Finally, the findings observed suggest the existence of new clues associated with the role that both abilities play in physicians and nurses. While the evidence demonstrates that empathy is an important aspect in the prevention of greater somatization and exhaustion among healthcare professionals at their workplace, lifelong learning is more beneficial for the reduction in work alienation and somatization when working responsibilities are shared. 

These findings also draw attention to the urgent need to introduce important changes in the organizational culture of healthcare institutions with characteristics similar as the one where this study was performed. It is evident that there are important structural issues that should be urgently resolved in healthcare institutions in LMICs, for example, moving forward from fragmented (and highly bureaucratized) healthcare systems to single or integrated ones and reinforcing primary care services with more healthcare professionals. However, in the meantime, other aspects related to professional abilities, such as empathy and lifelong learning, should be addressed in order to reduce the detrimental effect that work overload has on the health and wellbeing of workers with greater sense of professionalism.

## Figures and Tables

**Table 1:** Descriptive analysis and reliability coefficients.

Statistics	JSE	JeffSPLL	SCE	SS	SE	SA
Range possible	20–140	14–56	13–91	5–35	4–28	4–28
Range observed	73–128	37–56	13–91	5–35	4–28	4–28
Mean	88	50	65	24	22	19
Standard deviation (*SD*)	13	4	19	7	6	8
Quartile						
1st	80	48	54	20	19	13
2nd (Median)	82	50	71	25	25	22.5
3rd	89	53	79	30	27	24
Reliability	0.71	0.70	0.94	0.83	0.91	0.95

JSE, Jefferson Scale of Empathy; JeffSPLL, Jefferson Scale of physician lifelong learning; SCE, Scale of collateral Effects; SS, Scale of somatization; SE, Scale of exhaustion; SA, Scale of work alienation.

**Table 2:** Multiple linear regression models for somatization, exhaustion, and work alienation measures.

Dependent Variable	Predictors	*β*	*SE*	*t*	*p*
Somatization (SS)	Empathy (JSE)	−0.22	0.05	−4.55	<0.001
R^2^-adjusted = 0.29	Lifelong learning (JeffSPLL)	+0.59	0.17	+3.49	<0.001
*F*_(3,76)_ = 11.7; *p* < 0.001	Civil status (married)	−2.77	1.31	−2.12	0.037
Exhaustion (SE)	Empathy (JSE)	−0.07	0.03	−2.02	0.047
R^2^-adjusted = 0.72	Somatization (SS)	+0.44	0.07	+6.16	<0.001
*F*_(4,75)_ = 50.67; *p* < 0.001	Work alienation (SA)	+0.30	0.07	+4.58	<0.001
	Discipline (nursing)	−1.93	0.76	−2.53	0.01
Work alienation (SA)	Lifelong learning (JeffSPLL)	−0.39	0.15	−2.56	0.01
R^2^-adjusted = 0.61	Exhaustion (SE)	+1.00	0.09	+10.58	<0.001
*F*_(3,76)_ = 41.44; *p* < 0.001	Discipline (nursing)	+3.85	1.14	+3.37	0.001

JSE, Jefferson Scale of Empathy; JeffSPLL, Jefferson Scale of physician lifelong learning; SS, Scale of somatization; SE, Scale of exhaustion; SA, Scale of work alienation; *β*, beta coefficient; *SE*, standard error; *t*, t experimental; *p*, probability.

## Data Availability

The data are available upon request.

## References

[1] Karasek R.A., Theorell T. (1992). Healthy Work: Stress Productivity and the Reconstruction of Working Life.

[2] Viruez-Soto J., Delgado Bolton R.C., San-Martín M., Vivanco L. (2021). Inter-Professional Collaboration and Occupational Well-Being of Physicians Who Work in Adverse Working Conditions. Healthcare.

[3] Cotlear D., Gómez-Dantés O., Knaul F., Atun R., Barreto I.C.H.C., Cetrángolo O., Cueto M., Francke P., Frenz P., Guerrero R. (2015). Overcoming social segregation in health care in Latin America. Lancet.

[4] Atun R., de Andrade L.O.M., Almeida G., Cotlear D., Dmytraczenko T., Frenz P., Garcia P., Gómez-Dantés O., Knaul F.M., Muntaner C. (2015). Health-system reform and universal health coverage in Latin America. Lancet.

[5] Geoghegan V.S., Zavattiero C. (2012). Necesidades Básicas Insatisfechas (NBI): Una Aproximación a la Pobreza Estructural. Asuncion, Paraguay, Dirección General de Estadísticas, Encuestas y Censos (DEGEEC). https://www.ine.gov.py/Publicaciones/Biblioteca/documento/ebc6_Necesidades%20Basicas%20Insatisfechas%20(NBI).pdf.

[6] Giménez Caballero E., Rodríguez J.C., Ocampos G., Flores L. (2017). Composition of out-of-pocket health spending in the health system of Paraguay. Mem. Inst. Investig. Cienc. Salud.

[7] Pan American Health Organization/World Health Organization (2018). Evidence and Intelligence for Action in Health. Health Analysis, Metrics and Evidence. Health Situation in the Americas: Core Indicators 2018.

[8] Delgado-Maidana W., Vega-CArduz E., Sanabria L., Figueredo-Thiel S., Delgado Maidana W., Vega Carduz E., Sanabria L., Figueredo Thiel S.J. (2011). Prevalencia del síndrome de Burnout en médicos anestesiólogos del Paraguay durante el año 2010. Mem. Inst. Investig. Cienc. Salud.

[9] De Corbalán M.V., Ii S.M. (2013). Clima organizacional de enfermería en los hospitales regionales del Instituto de Previsión Social Organizational climate of the regional hospitals of the Social Security Institute. Mem. Inst. Investig. Cienc. Salud.

[10] Arnold L., Stern D.T., Stern D.T. (2006). What is medical professionalism?. Measuring Medical Professionalism.

[11] Arnold L. (2002). Assessing professional behavior: Yesterday, today, and tomorrow. Acad. Med..

[12] Fields S.K., Hojat M., Gonnella J.S., Mangione S., Kane G., Magee M. (2004). Comparisons of nurses and physicians on an operational measure of empathy. Eval. Health Prof..

[13] Hojat M. (2016). Empathy in Health Professions Education and Patient Care.

[14] Yuguero O., Ramon Marsal J., Esquerda M., Vivanco L., Soler-González J. (2017). Association between low empathy and high burnout among primary care physicians and nurses in Lleida, Spain. Eur. J. Gen. Pract..

[15] Soler-Gonzalez J., San-Martín M., Delgado-Bolton R., Vivanco L. (2017). Human connections and their roles in the occupational well-being of healthcare professionals: A study on loneliness and empathy. Front. Psychol..

[16] Marilaf Caro M., San-Martín M., Delgado-Bolton R., Vivanco L. (2017). Empathy, loneliness, burnout, and life satisfaction in Chilean nurses of palliative care and homecare services. Enferm. Clin..

[17] Hojat M., Erdmann J.B., Gonnella J.S. (2013). Personality assessments and outcomes in medical education and the practice of medicine: AMEE Guide No. 79. Med. Teach..

[18] Hojat M., Gonnella J.S. (2017). What Matters More about the Interpersonal Reactivity Index and the Jefferson Scale of Empathy? Their Underlying Constructs or Their Relationships with Pertinent Measures of Clinical Competence and Patient Outcomes?. Acad. Med..

[19] Schwan D. (2018). Should physicians be empathetic? Rethinking clinical empathy. Theor. Med. Bioeth..

[20] Hojat M., Spandorfer J., Louis D.Z., Gonnella J.S. (2011). Empathic and sympathetic orientations toward patient care: Conceptualization, measurement, and psychometrics. Acad. Med..

[21] Gleichgerrcht E., Decety J. (2013). Empathy in clinical practice: How individual dispositions, gender, and experience moderate empathic concern, burnout, and emotional distress in physicians. PLoS ONE.

[22] Thirioux B., Birault F., Jaafari N. (2016). Empathy Is a Protective Factor of Burnout in Physicians: New Neuro-Phenomenological Hypotheses Regarding Empathy and Sympathy in Care Relationship. Front. Psychol..

[23] San-Martín M., Delgado-Bolton R., Vivanco L. (2017). Professionalism and occupational well-being: Similarities and differences among latin american health professionals. Front. Psychol..

[24] Berduzco-Torres N., Medina P., San-Martín M., Delgado Bolton R.C., Vivanco L. (2021). Non-academic factors influencing the development of empathy in undergraduate nursing students: A cross-sectional study. BMC Nurs..

[25] Alcorta-Garza A., San-Martín M., Delgado-Bolton R., Soler-González J., Roig H., Vivanco L. (2016). Cross-Validation of the Spanish HP-Version of the Jefferson Scale of Empathy Confirmed with Some Cross-Cultural Differences. Front. Psychol..

[26] Berduzco-Torres N., Choquenaira-Callañaupa B., Medina P., Chihuantito-Abal L.A., Caballero S., Gallegos E., San-Martín M., Bolton R.C.D., Vivanco L., Delgado Bolton R.C. (2020). Factors Related to the Differential Development of Inter-Professional Collaboration Abilities in Medicine and Nursing Students. Front. Psychol..

[27] Hojat M., Veloski J., Nasca T.J., Erdmann J.B., Gonnella J.S. (2006). Assessing Physicians’ Orientation Toward Lifelong Learning. J. Gen. Intern. Med..

[28] Muliira J.K., Etyang C., Muliira R.S., Kizza I.B. (2012). Nurses’ orientation toward lifelong learning: A case study of Uganda’s national hospital. J. Contin. Educ. Nurs..

[29] Van Doorn Y., van Ruysseveldt J., van Dam K., Mistiaen W., Nikolova I. (2016). Understanding well-being and learning of Nigerian nurses: A job demand control support model approach. J. Nurs. Manag..

[30] López-Morales H., Rivera-Diaz E., Ore-Zuñiga A., Vera-Portilla A., San-Martín M., Delgado Bolton R.C., Vivanco L. (2020). Positive Impact of Professionalism on the Perception of Global Well-Being: A Study in Healthcare Professionals Starting Their First Working Experience in Peruvian Rural Areas. Front. Public Health.

[31] García-Sierra R., Fernández-Castro J., Martínez-Zaragoza F. (2016). Relationship between job demand and burnout in nurses: Does it depend on work engagement?. J. Nurs. Manag..

[32] Pisanti R., van der Doef M., Maes S., Meier L.L., Lazzari D., Violani C. (2016). How Changes in Psychosocial Job Characteristics Impact Burnout in Nurses: A Longitudinal Analysis. Front. Psychol..

[33] Siegrist J. (1996). Adverse health effects of high-effort/low-reward conditions. J. Occup. Health Psychol..

[34] Blanch J.M., Sahagun M., Cantera L., Cervantes G. (2010). Cuestionario de Bienestar Laboral General: Estructura y Propiedades Psicométricas. Rev. Psicol. Trab. Y Organ..

[35] Hojat M., Veloski J.J., Gonnella J.S. (2009). Measurement and correlates of physicians’ lifelong learning. Acad. Med..

[36] Veloski J.J., Hojat M., Stern D.T. (2006). Measuring Specific Elements of Professionalism: Empathy, Teamwork, and Lifelong Learning. Measuring Medical Professionalism.

[37] American Educational Research Association (AERA), American Psychological Association (APA), National Council on Measurement in Education (NCME) (2018). Estándares para pruebas educativas y psicológicas (M. Lieve, Trans.).

[38] Fritz C.O., Morris P.E., Richler J.J. (2012). Effect size estimates: Current use, calculations, and interpretation. J. Exp. Psychol. Gen..

[39] Tomczak M., Tomczak E. (2014). The need to report effect size estimates revisited. An overview of some recommended measures of effect size. Trends Sport Sci..

[40] Hojat M., Xu G. (2004). A visitor’s guide to effect sizes: Statistical significance versus practical (clinical) importance of research findings. Adv. Health Sci. Educ. Theory Pract..

[41] Cohen J. (1988). Statistical Power Analysis for the Behavioral Sciences.

[42] Bliese P. Multilevel: Multilevel Functions. https://cran.r-project.org/web/packages/multilevel/index.html.

[43] Kassambara A. Rstatix: Pipe-Friendly Framework for Basic Statistical Tests. https://cran.r-project.org/web/packages/rstatix/index.html.

[44] Navarro D. Lsr: Companion to “Learning Statistics with R”. https://cran.r-project.org/web/packages/lsr/.

[45] Gross J. Nortest: Tests for Normality. https://cran.r-project.org/web/packages/nortest/index.html.

[46] Hojat M., Gonnella J.S., Nasca T.J., Mangione S., Vergare M., Magee M. (2002). Physician empathy: Definition, components, measurement, and relationship to gender and specialty. Am. J. Psychiatry.

[47] Di Lillo M., Cicchetti A., Lo Scalzo A., Taroni F., Hojat M. (2009). The Jefferson Scale of Physician Empathy: Preliminary psychometrics and group comparisons in Italian physicians. Acad. Med..

[48] Suh D.H., Hong J.S., Lee D.H., Gonnella J.S., Hojat M. (2012). The Jefferson Scale of Physician Empathy: A preliminary psychometric study and group comparisons in Korean physicians. Med. Teach..

[49] San-Martín M., Roig-Carrera H., Villalonga-Vadell R.M., Benito-Sevillano C., Torres-Salinas M., Claret-Teruel G., Robles B., Sans-Boix A., Alcorta-Garza A., Vivanco L. (2017). [Empathy, inter-professional collaboration, and lifelong medical learning in Spanish and Latin-American physicians-in-training who start their postgraduate training in hospitals in Spain. Preliminary outcomes]. Aten. Primaria.

[50] Zahra Karimi F., Alesheikh A., Pakravan S., Abdollahi M., Damough M., Khosravi Anbaran Z., Amiri Farahani L. (2017). Surveying the factor structure and reliability of the Persian version of the Jefferson Scale of Physician Lifelong Learning (JeffSPLL) in staff of medical sciences. Electron. Physician.

[51] Gradiski I.P., Borovecki A., Ćurković M., San-Martín M., Delgado Bolton R.C., Vivanco L. (2022). Burnout in International Medical Students: Characterization of Professionalism and Loneliness as Predictive Factors of Burnout. Int. J. Environ. Res. Public Health.

[52] Hojat M., Gonnella J.S., Nasca T.J., Fields S.K., Cicchetti A., Lo Scalzo A., Taroni F., Amicosante A.M.V., Macinati M., Tangucci M. (2003). Comparisons of American, Israeli, Italian and Mexican physicians and nurses on the total and factor scores of the Jefferson scale of attitudes toward physician-nurse collaborative relationships. Int. J. Nurs. Stud..

[53] Fernández-Rodríguez L.J., Bardales-Zuta V.H., San-Martín M., Delgado Bolton R.C., Vivanco L. (2020). Empathy Enhancement Based on a Semiotics Training Program: A Longitudinal Study in Peruvian Medical Students. Front. Psychol..

[54] San-Martín M., Delgado-Bolton R., Vivanco L. (2017). Role of a semiotics-based curriculum in empathy enhancement: A longitudinal study in three dominican medical schools. Front. Psychol..

[55] Ramamurthy S., Er H.M., Devi Nadarajah V., Radhakrishnan A.K. (2019). Medical students’ orientation toward lifelong learning in an outcome-based curriculum and the lessons learnt. Med. Teach..

[56] Babenko O., Koppula S., Daniels L., Nadon L., Daniels V. (2017). Lifelong learning along the education and career continuum: Meta-analysis of studies in health professions. J. Adv. Med. Educ. Prof..

